# From HDAC to Voltage-Gated Ion Channels: What’s Next? The Long Road of Antiepileptic Drugs Repositioning in Cancer

**DOI:** 10.3390/cancers14184401

**Published:** 2022-09-10

**Authors:** Michele Pellegrino, Elena Ricci, Rosangela Ceraldi, Alessandra Nigro, Daniela Bonofiglio, Marilena Lanzino, Catia Morelli

**Affiliations:** Department of Pharmacy and Health and Nutritional Sciences, University of Calabria, 87036 Rende, Italy; michele.pellegrino@unical.it (M.P.); riccielena91@gmail.com (E.R.); rosy.ceraldi@gmail.com (R.C.); nigroale16@gmail.com (A.N.); daniela.bonofiglio@unical.it (D.B.)

**Keywords:** antiepileptic drugs, cancer, voltage gated sodium channels, voltage gated calcium channels, HDAC inhibitors, valproic acid, lamotrigine, carbamazepine, phenytoin, gabapentin, drug repositioning, adjuvant anti-cancer therapy

## Abstract

**Simple Summary:**

Although in the last decades the clinical outcome of cancer patients considerably improved, the major drawbacks still associated with chemotherapy are the unwanted side effects and the development of drug resistance. Therefore, a continuous effort in trying to discover new tumor markers, possibly of diagnostic, prognostic and therapeutic value, is being made. This review is aimed at highlighting the anti-tumor activity that several antiepileptic drugs (AEDs) exert in breast, prostate and other types of cancers, mainly focusing on their ability to block the voltage-gated Na^+^ and Ca^++^ channels, as well as to inhibit the activity of histone deacetylases (HDACs), all well-documented tumor markers and/or molecular targets. The existence of additional AEDs molecular targets is highly suspected. Therefore, the repurposing of already available drugs as adjuvants in cancer treatment would have several advantages, such as reductions in dose-related toxicity of combined treatments, lower production costs, and faster approval for clinical use.

**Abstract:**

Cancer is a major health burden worldwide. Although the plethora of molecular targets identified in the last decades and the deriving developed treatments, which significantly improved patients’ outcome, the occurrence of resistance to therapies remains the major cause of relapse and mortality. Thus, efforts in identifying new markers to be exploited as molecular targets in cancer therapy are needed. This review will first give a glance on the diagnostic and therapeutic significance of histone deacetylase (HDAC) and voltage gated ion channels (VGICs) in cancer. Nevertheless, HDAC and VGICs have also been reported as molecular targets through which antiepileptic drugs (AEDs) seem to exert their anticancer activity. This should be claimed as a great advantage. Indeed, due to the slowness of drug approval procedures, the attempt to turn to off-label use of already approved medicines would be highly preferable. Therefore, an updated and accurate overview of both preclinical and clinical data of commonly prescribed AEDs (mainly valproic acid, lamotrigine, carbamazepine, phenytoin and gabapentin) in breast, prostate, brain and other cancers will follow. Finally, a glance at the emerging attempt to administer AEDs by means of opportunely designed drug delivery systems (DDSs), so to limit toxicity and improve bioavailability, is also given.

## 1. Introduction

Cancer is one of the leading causes of death worldwide. Despite great advances have been made in cancer treatment, the current therapies (mainly chemotherapy, immunotherapy and targeted therapies) still have several drawbacks, such as limited efficacy, severe side effects and, not least, elevated costs [1], which are generally due to the high rate of failure of tested molecules in early stages clinical trials, but also to the expensive developing procedures. Therefore, repurposing of already approved drugs to treat off-label diseases might represent an attractive way to lower overall development costs and to shorten development steps [2].

Over the past 20 years, increasing evidences have suggested that various antiepileptic drugs (AEDs) exert anti-tumor activity, both in vitro and in vivo [3,4,5,6]. Since AEDs are commonly used in the symptomatic management of brain tumour-related epilepsy (BTE) [7,8] and of seizures in brain metastases from solid tumors [9], or else as analgesic against cancer-associated neuropathic pain [10,11], their potential antineoplastic effect has been reasonably questioned. However, while the mechanisms by which AEDs perform their anticonvulsant functions in epilepsy are well established, the molecular basis of their anti-cancer activity is still debated.

AEDs exert their anti-seizure action by interfering at different levels with the regulation of neuronal excitability [12], including inhibitory-GABAergic and excitatory-glutamatergic neurotransmission and, in particular, conductance through voltage-gated ion channels (VGICs), such as calcium, sodium, and potassium channels, which are essential for transmembrane signaling, ionic homeostasis and maintenance of membrane polarization [13]. In fact, blockade of the voltage-gated Na^+^ (VGSCs) [14,15] and Ca^++^ channels (VGCCs) [16,17] are the most common mechanism of action of currently available AEDs, since a reduced conductance of these channels is able to limit repetitive neuronal burst firing [18].

Interestingly, VGICs are functionally expressed in several types of carcinomas and VGICs-dependent membrane depolarization have been involved in cancer cell proliferation, invasion, and metastasis [19,20,21,22,23,24], suggesting that VGICs may be novel molecular targets for cancer treatment. In fact, voltage-gated K^+^ channels (VGPCs) can control cellular proliferation [25], while VGSCs, notoriously expressed in electrically excitable cells, including neurons and muscle cells, have been found upregulated in various cancers (e.g., prostate, cervical, breast cancer and others), where they seem to promote disease progression, favoring an invasive/metastatic phenotype [26]. Mainly three pore-forming isoforms of VGSCs, Na_V_1.5, Na_V_1.6 and Na_V_1.7, when functional (i.e., giving rise to sodium currents), have been involved in the invasive properties of carcinoma cells, where they seem to have a potential diagnostic, prognostic and therapeutic role [27,28]. Indeed, Na^+^ current has been reported to enhance invasion by promoting cysteine cathepsin activity in caveolae via allosteric regulation of the Na^+^/H^+^ exchanger type 1 [29], and Na_V_1.5 is a key regulator of a gene network that controls invasion [30]. An accurate review on the various Na_V_ subtypes abnormally expressed in cancer, together with the suggestion of targeting these channels by means of repurposing Na_V_-inhibitory drugs or new small inhibitory molecules, or even dietary interventions, as promising anti-cancer strategies has been recently reported by Lopez-Charcas et al. [31].

Similarly, several members of the VGCCs family, i.e., the group of high voltage-gated channels Ca_V_ 1 (L-type) plus the three subtypes of Ca_V_ 2 (P/Q-, N- and R-type) channels, and the group of low voltage-gated channels, the Ca_V_ 3 (T-type), have been involved in tumorigenesis and cancer progression, being highly expressed in most types of cancer [32,33]. In particular, T-type VGCCs are aberrantly expressed and often deregulated in cancer cells, supporting their proliferation, survival and resistance to treatments [34], so they were proposed as attractive molecular targets for anticancer therapy [35,36]. Several recent reviews give an accurate overview on VGCCs, their classification, pharmacology and proposed mode of action in cancers [37,38,39].

In line with these observations, AEDs, acting as Na^+^ and Ca^++^ channels blockers, have been shown to inhibit cancer cell proliferation, invasion, tumor growth and metastasis in preclinical models [36,40] and their use has been inversely associated with cancer risk in colorectal, lung and gastric cancers as well as hematological malignancies [41].

In addition, the anti-proliferative effect of several AEDs on cancer cells has also been ascribed to their ability to inhibit histone deacetylases (HDACs) [42]. Histone acetylation (a transcription-activating modification) and deacetylation (associated with condensed chromatin and, therefore, with transcriptional repression) are regulated by histone acetyltransferases (HATs) and HDACs, respectively [43]. HDACs are often dysregulated in numerous diseases, including cancer [44], where they promote tumorigenesis by inducing chromatin condensation and transcriptional repression of tumor suppressor (TS) genes. Thus, reactivation of TS genes transcription by means of HDAC inhibitors (HDACi) treatment might be responsible for the observed arrest in cell growth, block in cell cycle progression and induction of apoptosis, although HDACi may also trigger other molecular events, including generation of reactive oxygen species (ROS) and inhibition of angiogenesis [45]. Extensive literature and numerous reviews continuously offer interesting and deep insight into HDACs classification, activity, role in cancer and therapeutic implications [46,47,48,49].

**Figure 1:**
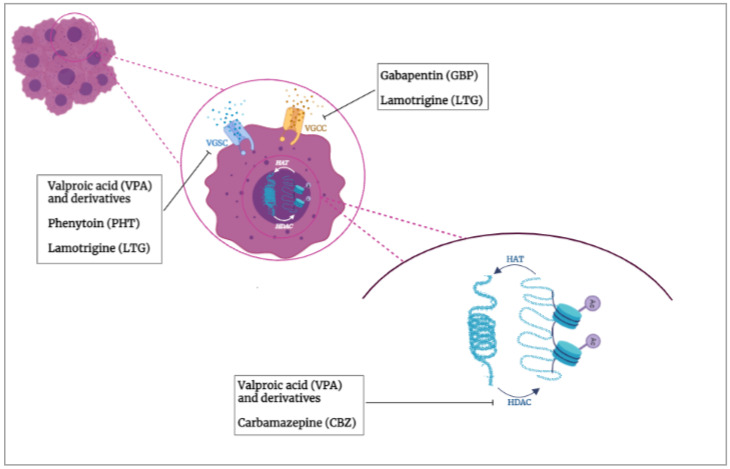
AEDs molecular targets. VGSCs and/or VGCCs are emerging hallmarks of invasive cancers and, together with HDACs, have been proposed as therapeutic targets for most of the commonly used AEDs.

Therefore, this review is rather aimed at highlighting the role that several AEDs play in breast, prostate and other types of cancers, mainly focusing on their ability to block voltage-gated Na^+^ [50] and Ca^++^ channels [35,36], as well as on their HDACi properties [51] (Figure 1). AEDs potential as adjuvants in cancer treatment and the advantages of drug repositioning will be discussed as well.

## 2. HDACs and VGICs Potential Functional Interaction in Cancer

As more accurately reviewed by Renzini et al. [52] and Bahl and Seto [53], to date, 18 different mammalian HDACs have been identified and divided into four classes (from Class I to IV). Most Class I and Class III (sirtuins) HDACs are ubiquitously expressed and primarily localized in the nucleus, with the exception of SIRT2 (mainly found in the cytosol) and SIRT3, -4, and -5 that are present exclusively in mitochondria). Class II HDACs are characterized by tissue-specific expression and stimulus-dependent nucleus-to-cytoplasm shuttling, while HDAC11, the only member of Class IV, seems to be mainly expressed in the kidney, heart, brain, skeletal muscle, and testis, and is localized in the nucleus of the cell.

HDACs deacetylate histones and result in chromatin condensation and epigenetic repression of gene transcription. However, HDACs also catalyse the deacetylation of many non-histone proteins, including signal transducers and transcription factors, thus leading to changes in the transcriptome and cellular signalling ([54] and references therein). Interestingly, epigenetic modifications by HDACs seem to represent an important regulatory mechanism underlying VGICs gene expression, and HDAC inhibitors have been reported to modulate their expression and/or activity, including voltage-gated sodium [55] and potassium channel [56,57].

Noteworthy, in cancer cells HDACi seem to enhance the expression of VGICs, thus promoting tumor progression. Conversely, in the heart tissue, SIRT1 has been reported to deacetylate Na_V_1.5 at lysine 1479 (K1479), stimulating inward depolarizing cardiac Na+ current (I_Na_) via lysine-deacetylation-mediated trafficking of Na_V_1.5 to the plasma membrane [58]. Moreover, pan-HDACi have been shown to decrease peak I_Na_ density, without significantly altering SCN5A mRNA levels, but strongly reducing Na_V_1.5 protein levels [59,60].

However, a work by Jang and Jeong [61], showed that the expression of the ion channel marker genes, e.g., SCN5A, KCNA4, and CACNA1G, was highly increased following treatment with HDACi; however, the expression of others was either decreased or unchanged.

Therefore, although a cooperative functional interaction between HDACs and VGICs in cancer cells might help explaining the anti-cancer efficacy of some AEDs for their ability of targeting both sodium and/or calcium channels and HDACs, caution and further investigantions are still needed before claiming such an assumption.

## 3. AEDs in Breast Cancer

### 3.1. VGICs and HDACs as Prognostic Markers and Therapeutic Targets in Breast Cancer

Breast cancer (BC) is the most widespread cancer in women. It is a heterogeneous and complex pathology, whose treatment of choice is based on the histological characteristics of the tumor [62]. Despite significant progress in the treatment of BC, serious adverse effects, high toxicity to normal cells, and the occurrence of multi-drug resistance (MDR), including that to antiestrogens, still limit the efficacy of therapy of BC patients. Thus, new agents with improved effectiveness and decreased resistance compared to currently used treatments are highly needed. Accumulating evidences are revealing that the voltage-gated ion channels play an important role not only in the excitable cells, but also in non-excitable cells, such as epithelial cells such as breast cancer cells (BCCs). Heterogeneous bioelectricity can promote not only the initiation, but also the proliferation and metastasis of BCCs and it may be caused by differential expression of ion channels [63]. In this context, VGSCs have been involved in the growth and invasiveness of BCCs [64,65]. Particularly, the VGSCs subunit Na_V_ 1.5 (to date, nine α-subunits, Na_V_ 1.1–1.9 and four β-subunits, β1–4, have been identified in mammals [66]) is the most highly expressed α subunit in breast [29,65,67], and it associates with poor prognosis in clinical BC specimens [68], suggesting that VGSCs may have utility as an additional prognostic markers for BC progression [64,69].

Similarly, the expression of calcium channel transcripts has been highlighted as a potential biomarker of certain types of cancer, including breast, where different VGCCs family members have been found aberrantly expressed and deregulated [32]. For instance, the three VGCCs Ca_V_ 1.3 (L-type), Ca_V_ 3.2 and Ca_V_ 3.3 (T-type) have been reported to promote cell proliferation and tumor growth in BC and Ca_V_ 3.2 isoform antagonists have shown anti-proliferative and cytotoxic effects [70,71]. Moreover, amplification of the *CACNG4* gene, encoding for an L-type VGCC γ subunit, has recently been found in BC with poor prognosis, where deregulated calcium influx and signaling has been associated to increased BCCs survival and metastasis [72]. Accordingly, MCF-7 and MDA-MB-231 BCCs are characterized by a more depolarized resting membrane potential respect to normal mammary epithelial cells and depolarization-induced calcium influx was hypothesized as a requirement for the growth of BCCs. In fact, calcium removal from culture medium or the use of verapamil, a VGCCs and VGSCs [73] blocker clinically employed in the treatment of hypertension and coronary disease, inhibited BCCs growth, inducing apoptosis [19]. Nevertheless, the VGCC isoform Ca_V_ 3.1 has been shown to act as a tumor suppressor gene in BCCs, retarding proliferation and enhancing apoptosis [74].

Therefore, an accurate investigation of VGSCs and VGCCs isoforms distribution within BC molecular subtypes, might give important information for the development of effective therapeutic strategies in tumors with aberrant sodium and calcium signaling. So far, several compounds that are able to inhibit the sodium current and the calcium influx have been already reported to effectively reduce BCCs growth and progression [50,67,68,75]. Among these, AEDs such as phenytoin (PHT), valproic acid (VPA) and lamotrigine (LTG) seem to be promising therapeutic tools in the management of BC. In fact, as mentioned, some of them do not only act as VGICs blockers, but also as epigenetic modulators (mainly HDACi) which showed great potential in significantly reducing tumor malignancy when used in combination to standard chemotherapy regimens [76]. Notably, the use of AEDs in combination to radiation therapy was also associated to an improved overall survival (OS) in BC patients with brain metastases [77].

### 3.2. Valproic Acid (VPA)

The VGSC blocker valproic acid (2-propylpentanoic acid, VPA), a short chain carboxylic acid, is a well-known anticonvulsant, whose anticancer activity has been mainly ascribed to its epigenetic modulator properties [78].

The effect of VPA, either used alone or in combination with other anti-cancer agents, on different histological subtypes of BC has been recently and nicely reviewed by Wawruszak A. et al. [79]. VPA is a clinically available HDACi which has been reported to inhibit proliferation, cell cycle, survival, cell migration, and hormone receptor expression of BCCs in both the pre-clinical and clinical settings [42]. VPA has an antiproliferative action in estrogen receptor alpha positive (ERα+) BCCs, inducing apoptosis through the activation of caspases 9 and 8 and increasing p21, thus leading to cell cycle arrest [80]. Interestingly, VPA is also able to restore cell functions silenced by epigenetic modifications. In ER-negative (ER-) BCCs, MDA-MB-231, VPA confers an estrogen-sensitive phenotype and, consequently, a sensitivity to antiestrogen treatment [81]. In addition, VPA has been shown to inhibit the migration of MDA-MB-231 BCCs through the upregulation of the metastatic suppressor NM23H1 [82].

Several studies have analyzed the efficacy of VPA action used in combination with other well-known antineoplastic agents. Capecitabine is an oral prodrug of 5-fluorouracil (5-FU), which is commonly used in the treatment of metastatic BC. To work properly, capecitabine requires high levels of thymidine phosphorylase (TP), a key enzyme that allows its conversion into 5-FU within the tumor. HDACi, including low concentrations of VPA, induce dose- and time-dependent upregulation of mRNA levels and TP protein expression in BCCs, but not in the non-tumorogenic MCF-10A cells. The combined treatment with capecitabine and VPA, appears to have synergistic/additive antiproliferative and pro-apoptotic effects in MCF-7, SKBR3 and MDA-MB-468 cells, and only additive in MDA-MD-231 BCCs but not in TP knockdown cells, both in vitro and in vivo [83].

Another study showed that combinations of cisplatin, or cis-diamminedichloroplatinum (II) (CDDP) with VPA or another HDACi, suberoylanilide hydroxamic acid (SAHA, vorinostat), have an additive effect in MCF-7, whereas in T47D cells both combinations had a synergistic effect. On the other hand, a sub-additive (antagonistic) interaction was observed in MDA-MB-231, for CDDP/VPA, and an additive effect for CDDP/SAHA combinations. Importantly, the HDACi/CDDP treatment resulted in increased apoptosis and cell cycle arrest in all tested cell lines compared to single therapy, suggesting that HDACi could be combined with CDDP to optimize treatment regimen in some human breast cancers [84]. Of note, since epigenetic alterations play a fundamental role in stemness, inhibition of HDACs by VPA results in BC stem cells (BCSCs) apoptosis, although the phenomenon occurs less markedly than in BCCs [85].

#### 3.2.1. VPA Derivatives

VPA derivatives seem to be even more efficacious than VPA on BCCs and show a better safety profile.

Several compounds have been described. The VPA aryl-derivative N-(2-Hydroxylphenyl)-2-propylpentanamide was less hepatotoxic than VPA, being as efficacious as VPA in killing cancer cells, but at lower doses [86]. A VPA prodrug AN446 was about 60 times more powerful than VPA in killing cancer cells and in inhibiting in vitro migration and invasion. AN446 also exhibited high selectivity and HDAC inhibitory activity in cancer cells and low in noncancerous cells, compared to other HDACi. In triple negative MDA-MB-231, AN446 and VPA acted in synergy with doxorubicin, thus reducing its dose-dependent toxicity [87].

More recently, poorly water-soluble aryl-VPA derivatives compounds targeting the HDAC8 enzyme were conjugated to four generations Polyamidoamine (G4 PAMAM) dendrimers, employed as drug carriers, which were able to deliver the drug to several BCCs, where they could exert their cytotoxic activity [88]. VPA and its derivative HPTA, especially in combination, have been also reported to potentiate radiotherapy (RT) treatment, by effectively sustaining an activated anti-tumor immune response. Indeed, both compounds are able to (a) induce myeloid-derived macrophages, polarizing them toward the M1 phenotype, (b) increase activated CD8+ T cells, and c) inhibit angiogenesis. These effects do translate in the reduction of tumor growth and a much lower probability of tumor recurrence [89].

#### 3.2.2. Clinical Trials

The effects of VPA or its derivatives in combination therapy with conventional chemotherapeutics drugs were determined in several clinical studies (reviewed in [42,79]).

One of these reports the administration of VPA in combination with epirubicin in phase I, or FEC100 (5-FU, epirubicin, and cyclophosphamide), an approved regimen for BC patients, in Phase II. Interestingly, sustained plasma concentrations of VPA exceeding those required for in vitro synergy were achieved with an acceptable toxicity profile and antitumor efficacy [90].

Another clinical trial monitored the efficacy and the safety of co-administration of the combination of the VPA derivative magnesium valproate and the methyltransferase inhibitor hydralazine to neoadjuvant doxorubicin and cyclophosphamide in locally advanced breast cancer. The study showed that therapy with valproate and hydralazine is safe and seems to increase the efficacy of conventional chemotherapeutic agents, reaching a complete response in 31% of patients [91].

A third, recently ended, study was conducted to determine the anti-tumor efficacy and the safety profile of bevacizumab and temsirolimus alone or in combination with VPA or cetuximab in patients with advanced or metastatic malignancy, including BC [92]. No results were posted so far.

### 3.3. Phenytoin (PHT)

PHT is an AED and an antiarrhythmic (class 1b), which, preferentially binding to the inactive state of VGSCs, enhances steady-state inactivation and, consequently, inhibiting the sodium current [93], which is involved in migration and invasion of tumor cells [14,94]. Firstly synthesized in 1908, its anticonvulsant role was only discovered in 1937. Since then, PHT was proved to have many other clinical properties, very likely, due to the existence of multiple targets, besides VGSCs. PHT has been explored in over 100 different disorders and, among these, breast cancer together with optic neuritis seem to be the last two promising clinical indications.

As recently recalled by Keppel Hesselink and Kopsky [95], Yang M. and co-workers showed how PHT, used at clinically achievable concentrations, is able to suppress Na^+^ current in highly metastatic MDA-MB-231 BCCs expressing functional VGSCs (especially Na_V_ 1.5), thus inhibiting VGSC-dependent migration and invasion. On the other hand, PHT had no effect on the weakly/non-metastatic MCF-7 BC cell line that do not express any functional VGSC. The same authors also reported that *SCN5A* (encoding for Na_V_ 1.5) is up-regulated in BC samples in several datasets, and associates with poor prognosis, favoring an invasive/metastatic phenotype [68]. This observation is in line with previous published data from Fraser S.P. et al., who reported that Na_V_ 1.5 expression is significantly up-regulated in metastatic human BCCs and tissues, and that its activity potentiates cellular motility and invasion in vitro and correlates with BC progression in vivo, including clinically assessed lymph node metastasis [64]. Accordingly, Nelson M. et al. showed that PHT reduced breast tumor growth, invasion and metastasis in vivo [40].

Therefore, repurposing the existing VGSC-blocking therapeutic drug PHT as a potential new strategy to improve patient outcomes in metastatic BC would be desirable. Of note, its use in the treatment of ERα+ BC patients does not seem to be appropriated, both for the lack of functional VGSCs in this subset of tumors and for the SERM activity (either strong antagonist or weak agonist) exerted by PHT on ERα [96]. Despite this, a Phase II clinical trials sponsored by Georgetown University started in 2021 with the scope of evaluating the efficacy and safety of the mTOR inhibitor SM-88 (a modified dysfunctional tyrosine) in combination with three subtherapeutic conditioning agents (PHT, methoxsalen and sirolimus, i.e., rapamycin, another mTOR inhibitor), in patients with metastatic HR+/HER2- BC [97]. No results were published yet.

### 3.4. Lamotrigine (LTG)

LTG is an anticonvulsant used in epilepsy and in bipolar disorder. Similarly to other AEDs, LTG works by blocking voltage-dependent calcium (N- and P/Q/R-types, T-type weakly if at all, no action on the L-type) [16,17,98,99] and sodium channels [14,15,100,101]. However, additional targets could be responsible for different mechanism of action, which, in turn, could lead to novel therapeutic use of the drug. For example, LTG seems to share with other AEDs the HDACs inhibitory activity. In fact, LTG has been reported to exerts a neuroprotective effect against glutamate-induced excitotoxicity in rat cerebellar granule cells (CGCs) by increasing the levels of acetylated histone H3 (AcH3) and H4 (AcH4), which likely involves histone deacetylase inhibition [102]. Similarly, treatment with LTG or other mood stabilizers induced significant increases in AcH3, HDAC2, HDAC3 and HDAC5 in different brain areas of an experimental mouse model, supporting the possibility that antidepressant-like effects involve epigenetic modifications associated with changes in HDAC expression [103].

The histone deacetylase inhibition function has never been reported for LTG in breast cancer. However, we recently showed the anti-proliferative effect of LTG in BCCs and inhibition of breast tumor growth in animal models. LTG treatment led to a marked reduction of AKT phosphorylation and its direct downstream targets FoxO3a and GSK-3β in both ERα+ MCF-7 and ERα- MDA-MB-231 BCCs, thus LTG effect does not depend on the ERα status, but seems to occur through the induction of PTEN and FoxO3a mRNA and protein expression [104]. Considering that LTG is a VGCC blocker and that Ca^++^ influx has been reported to activate the PI3K/Akt axis in different cell systems [105,106,107], our results well fit with previously published reports showing that T-type Ca++ channel inhibition is able to interfere with mTOR/AKT pathway in a human lung adenocarcinoma cell line [108] and disrupted Akt signaling, promoting apoptosis, in glioblastoma cells [109].

**Figure 2:**
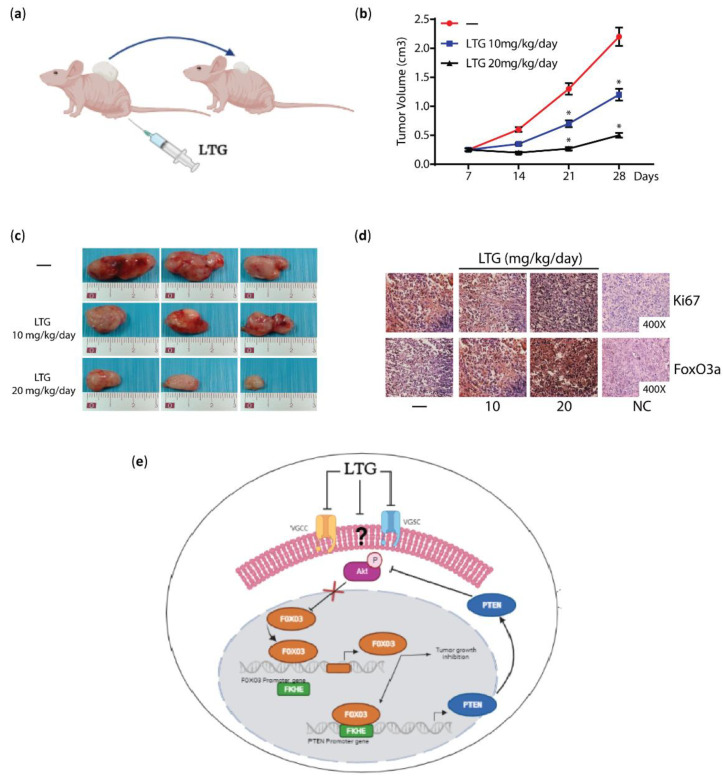
LTG inhibits BC tumor growth by inducing FoxO3a expression. (**a**) Mice bearing BCCs-derived xenografts were treated with LTG at 10 mg/kg/day and 20 mg/kg/day. (**b**) Tumor growth was monitored by caliper, measuring the visible tumor sizes at indicated time points. *, *p* < 0.05 versus control. (**c**) At the end of experiment, tumors were explanted and representative images are shown. (**d**) Ki67, a marker of proliferation index, and FoxO3a expression was evaluated in FFPE sections of tumor xenografts deriving from mice treated or not (-) with LTG. NC, negative control. (**e**) Schematic representation of LTG hypothetical mechanism of action in BC. LTG, by presumably blocking VGSCs and/or VGCCs or an unknown target, inhibits AKT signaling, activating its target FoxO3a, which induces its own transcription and expression and, consequently, the expression of its downstream target, PTEN, which, in turn, maintains inactive the PI3K/AKT pathway, thus sustaining an antiproliferative autoregulatory loop. Results and schemes shown here are adapted from Pellegrino M. et al. [104].

Notably, we also observed that LTG is able to restore the response to antiestrogen treatment in MCF-7 derived Tamoxifen resistant cells (TamR) both in vitro and in vivo, by inducing the expression of FoxO3a, whose levels are significantly downregulated in TamR. LTG-mediated FoxO3a re-expression was accompanied by a strong reduction of tumor mass in TamR-derived mouse xenograft, suggesting how LTG might represent a valid candidate in combination therapy to prevent resistance to tamoxifen in BC treatment [110] (Figure 2).

Interestingly, ongoing studies conducted in our laboratory on MCF-7 BCCs, unveiled a landscape of potential additional molecular targets for LTG, which are currently under investigation. Results from this study will be included in an upcoming publication.

At present, no clinical studies employing LTG, neither alone nor in co-administration with other chemotherapeutics, have been approved on BC patients.

### 3.5. Carbamazepine

Carbamazepine (CBZ) is a well-known AED in use in clinical practice since 1962. As other AEDs, it acts as an HDACi [111] with anti-cancer properties. In fact, it showed interesting anti-metastatic potential in BCCs by inducing the proteasomal degradation of the tyrosine kinase receptor HER2 and, consequently, inhibiting BCCs proliferation [112]. Mechanistically, CBZ, through the inhibition of HDAC6, promoted the acetylation of heat-shock protein 90 (Hsp90), disrupting its role of chaperone for the HER-2 oncoprotein, which, therefore, undergoes degradation. Another proposed role for CBZ in metastatic BC is its ability to significantly inhibit the formation of “circular chemorepellent-induced defects” (CCIDs), generated in vitro through a three-dimensional (3D) co-culture model consisting of MCF-7 BCC spheroids and of hTERT-immortalised lymph endothelial cell (LEC; derived from foreskin) monolayers, which mimics the tumour spheroid-induced prometastatic intravasation gates in the lymph endothelial cell barrier. The inhibition of CCIDs formation correlated with the inhibition of NF-κB, involved in cell motility, and with the inactivation of the mobility proteins MLC2, MYPT1 and FAK which are necessary for LEC migration [113].

## 4. AEDs and Prostate Cancer

### 4.1. Prostate Cancer: Diagnostic Markers and Therapeutic Management

Prostate cancer (PCa) is the second most commonly diagnosed cancer and the fifth leading cause of cancer death among men worldwide [114]. Although emerging evidence points to a key role of a several gene mutations in PCa pathogenesis and progression, the androgen receptor (AR) signaling is still the focus for the screening of new therapeutics, including novel androgen deprivation approaches. In fact, androgen deprivation therapy (ADT) represents a backbone of treatment for patients with advanced disease, while localized PCa is generally managed by deferred treatment or active local therapy (such as radical prostatectomy or radiation therapy), with or without ADT [115]. However, due to advances in understanding genomic landscapes and biological functions, the treatment of PCa continues to evolve. Therefore, next-generation AR signaling inhibitors, bone-targeting agents and poly(ADP-ribose) polymerase (PARP) inhibitors, agents targeting other oncogenic signaling pathways, such as cyclin-dependent kinase (CDK)4/6, AKT, wingless-type protein (WNT), and epigenetic marks, have successively entered clinical trials [116].

Targeted therapies against newly identified tissue-specific proteins, such as the prostate-specific membrane antigen (PSMA), a transmembrane glycoprotein, whose levels gradually increase from normal epithelium to PCa, have been also proposed as promising theranostic agents that could improve both diagnostic accuracy and therapeutic efficacy [117].

Furthermore, epigenetic aberrations, including changes in DNA methylation patterns and/or histone modifications, have been recognized as key drivers of prostate carcinogenesis. However, epigenetic modifications are reversible and numerous epigenetic modulating compounds were reported to be effective in PCa growth control and are being tested in pre-clinical and clinical trials as potential therapeutic agents for PCa management [118]. Among these, PCa-specific long non-coding RNAs (lncRNAs) have been reported to play an important role in PCa tumorigenesis, thus they have been proposed as novel biomarkers for early diagnosis and prognosis of metastatic or recurrent PCa, as well as a therapeutic targets [119]. Moreover, epigenetic modifiers such as HDACi have been qualified as an attractive candidate class to be employed in PCa combination therapy [120]. Finally, similar to other types of cancer, some VGICs, including VGSCs and VGCCs, are also abnormally expressed in PCa and correlate with a poorer prognosis [121,122,123].

As already discussed for BC, AEDs, for their dual activity as both HDACi and VGIC blockers, could represent a valuable therapeutic strategy for PCa, as well. An overview of the main findings on the anti-tumor activity exerted by several AEDs on PCa cells and animal models follows.

### 4.2. VPA and Derivatives in PCa

At present, only two recent, but not exhaustive, reviews focus on the role of VPA, either used alone or in combination with other molecules, on PCa development, growth and progression [124,125]. Therefore, a more detailed overview of the main findings on the antitumor activity exerted by this specific AED on PCa follows.

#### 4.2.1. VPA Anti-Proliferative Activity

The anti-proliferative effect of VPA was firstly observed in LNCaP human PCa cells, where, by acting as histone deacetylases inhibitor, it caused the down-regulation of the well-established PCa hallmark prostate-specific antigen (PSA), as well as the up-regulation of pro-apoptotic caspase-3, of the tissue inhibitor of matrix metalloproteinase-3 and the insulin-like growth factor binding protein-3 [126]. VPA-mediated increase in histone H3 acetylation and in caspase-2 and caspase-3 activation was confirmed in both AR-positive (LNCaP and C4-2) and in AR-negative (DU145 and PC3) PCa cells, where even lower doses of chronically administered VPA (10–14 days) resulted in a marked decrease in cell proliferation rate and in a significant reduction of tumor xenograft growth in vivo [127].

Interestingly, since HDCAi, by changing the acetylation status of histones, can loosen the chromatin structure, dose- and time-dependent changes in nuclear structure have been described in VPA-treated PCa cells as well as in the deriving tumor xenografts and in the drug-filtering organs, liver and kidney, in vivo, proposing the nuclear structural alterations as potential biomarkers for histone deacetylase inhibitor treatment [128].

In AR-positive cells, VPA-dependent growth suppression seems to correlate with the increase in cellular prostatic acid phosphatase (cPAcP) expression, a unique prostate-specific tumor suppressor, whose loss is associated with prostate carcinogenesis [129]. On the other hand, a Fas-dependent apoptosis associated with Fas and Fas ligand overexpression has been described mainly for less differentiated, AR-negative PC3 and DU145, cell models of advanced, clinically untreatable stages of PCa progression [130].

However, multiple other mechanisms have been proposed for VPA-dependent growth inhibition of PCa cells both in vitro and in vivo, including cell cycle arrest (decreases the S phase and Cyclin A [131], increase in Cyclin D2 [132], p21 and p27 [131] and decrease in PCNA, cyclin D1 and AR expressions have been reported), apoptosis, autophagy [133] (through the Akt/mTOR pathway inactivation [134]), senescence, and angiogenesis inhibition [135,136]. The suppression of tumor angiogenesis was associated to Thrombospondin1 (a mediator of cell-to-cell and cell-to-matrix interactions), TIMP (a MMPs inhibitor) and TGFβ upregulation, and to IGF1 and VEGF down-regulation [137,138].

Finally, considering that lipid metabolism reprogramming is now being recognized as an emerging hallmark of cancer, VPA has been recently reported to exert its inhibition on PCa cell growth by reducing lipogenesis, by targeting the C/EBPα/SREBP-1 pathway [139].

#### 4.2.2. VPA in PCa Progression

Several studies demonstrate how VPA is also effective in blocking PCa progression. In fact VPA has been shown to inhibit PCa cell migration, by increasing the expression of E-cadherin (E-cad), a key protein in cell-cell adhesion, EMT, cancer cell migration and invasion, whose loss is often associated with PCa metastatic events [140,141]. The upregulation of the metastasis suppressor protein N-myc Downstream Regulated Gene-1 (NDRG1) has been also reported, under VPA treatment, in highly metastasizing PC3 cells [142]. On the other hand, EMT inhibition and metastasis suppression in VPA-treated PCa cells was accompanied by the down-regulation of SMAD4, a key molecule in TGF-β-induced EMT [143]. The decrease occurred both at the mRNA and protein level, and was paralleled by an increase in mono-ubiquitinated SMAD4 [144], very likely as a result of VPA-dependent induction of the Transcriptional Intermediary Factor 1γ (TIF1γ), a vital protein molecule that possesses ubiquitination enzyme activity [145]. All these studies suggest that VPA treatment, combined with specific SMAD4 inducers, can form the basis for a novel PCa treatment. A deeper discussion on the VPA suppressive role on TGF-β signaling in PCa therapy has been included in a recent review [125].

In a recent paper from Chen et al. [146], through an accurate analysis of publicly available microarray datasets (Gene Expression Omnibus database), HCG18 and MCM3AP-AS1 lncRNAs have been proposed as novel biomarkers of PCa progression, since they resulted in being positively associated with bone metastasis, increased abundance of M2 Macrophage infiltration and poor prognosis. Interestingly, by querying the Connectivity Map (CMap) and the Comparative Toxicogenomics Database (CTD), HDACi VPA and trichostatin A were predicted as potentially effective for the treatment of PCa bone metastasis by targeting both the lncRNAs, thus reversing the high expression of HCG18 and MCM3AP-AS1 and reducing the number of HCG18- and MCM3AP-AS1-mediated M2 type macrophages) [146].

**Figure 3:**
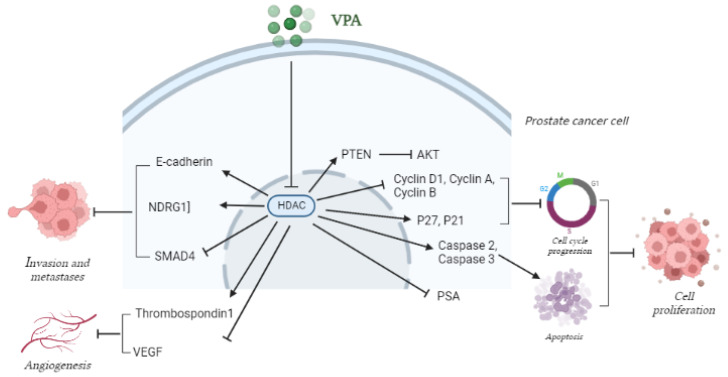
VPA anti-tumor activity in PCa. VPA inhibits HDACs activity. The deriving histone acetylation results in the induction or inhibition of several molecular targets, which are involved in PCa proliferation and progression, as well as in angiogenesis.

A schematic representation of the main VPA-induced biological effects in PCa and the involved molecular targets is reported in Figure 3.

#### 4.2.3. VPA and Neuroendocrine Transdifferentiation (NET) of PCa Cells

PCa originates as an androgen-dependent hyper-proliferation of the epithelial prostate cells, and it evolves in an androgen-independent, highly aggressive cancer. NET plays an important role in the progression of PCa to an androgen-independent state, mainly related to advanced disease and poor clinical outcome. In cancer cells, VPA has been reported to act as a differentiation agent, by inducing the expression of neuron-specific markers enolase and β-III Tubulin [131], a decrease in PSA, AR, androgen receptor coregulator (ARA24) expression and an up-regulation of some of the UDP-glucuronosyltransferases (UGT2B11 and UGT2B7) implicated in dihydrotestosterone (DHT) catabolism, all leading to an altered response to androgen therapy [147].

The VPA-mediated NET process was also paralleled by PPARgamma activation, and the use of a specific PPARgamma antagonist was able to significantly reduce the expression of NE markers induced by VPA. Although PPARgamma inhibition has been suggested as a suitable adjuvant treatment strategy in PCa [148], concerns still exist about the potential use of VPA in PCa therapy.

However, VPA-induced NET was generally described as an early event in cultured PCa cells, although associated with a reduction in overall cell proliferation. Moreover, Sidana et al. did not reveal any induction of the NET markers CgA, synaptophysin or NCAM in VPA-treated PCa xenografts, i.e., in a physiologically relevant in vivo setting [149]. Therefore, additional studies are needed to clarify this controversial issue.

#### 4.2.4. VPA in Combination Therapy for PCa Treatment

##### Combination of VPA and the Mammalian Target of Rapamycin (mTOR) Inhibitors in PCa

The mammalian target of rapamycin (mTOR) is elevated in PCa, making this protein attractive for tumor treatment. Wedel and co-workers were the first to test the antitumor activity of the mTOR inhibitor RAD001 (Everolimus)-VPA combination on PCa cell lines. Although separate application of RAD001 or VPA reduced tumor cell growth and impaired cell cycle progression, a significant additive effect was observed when both drugs were used together, with cell-cycle-regulating proteins cdk1, cdk2, cdk4 and cyclin B strongly reduced and p21 and p27 increased. EGF-R and ERK1/2 signals were decreased as well [150]. The same authors also demonstrated that separate application of RAD001 or VPA reduced tumor cell adhesion, migration and invasion. Additive effects were observed on the migratory and invasive behavior but not on tumor–endothelium and tumor–matrix interactions when both drugs were used in combination [151].

Similar synergistic effects of VPA in combination with another mTOR inhibitor, temsirolimus, were reported on LNCaP cells, compared to the single treatments. The drugs’ combination resulted in a decrease in cell proliferation, which was accompanied by a significant upregulation of insulin-like growth factor-binding protein-3 (IGFBP-3), a mediator of apoptosis. Oddly, no differences in tumor growth have been observed in vivo among the single or the combination treatments [152].

VPA was also able to counteract temsirolimus resistance, reducing cell growth, very likely by downregulating Cdk1, cyclin B, active mTOR and the mTOR sub-complex Raptor [153]. In addition., VPA treatment reduced tumor cell–matrix interaction, chemotaxis and the migration of highly motile temsirolimus-resistant PCa cells, which also expressed reduced levels of integrin α5 [154].

##### Combination of VPA and the Hypoglycemic Drug Metformin (MET) in PCa

Accumulating evidence, reviewed in [124], shows that MET and VPA repurposing, either alone or in combination, could potentially play a role in slowing down PCa progression. In PCa cells, as well as in patient-derived prostate tumor explants, MET/VPA combination synergistically inhibited proliferation and induced apoptosis in a P53- and AR-dependent manner, without significantly affecting normal Prostate Epithelial Cells (PrEC) [155]. The higher efficacy and low toxicity of MET/VPA combination compared to either drug alone has been confirmed in mouse xenografts [156].

##### Other Proposed VPA Combination Therapies in PCa

Various other drug combinations have been proposed for the treatment of PCa. A synergistic association was observed using VPA together with gossypol (GOS), a BH3 mimetic, as a sensitizing co-therapy to radiation and chemotherapy in metastatic PCa treatment. VPA enhanced the cytotoxicity of GOS on DU145 PCa cells, culminating in increased DNA damage (also due to downregulation of proteins involved in DNA-repair) and cell death [157]. Similarly, a selective targeting of homologous recombination (HR) repair pathways, leading to decreased expression levels of DNA-repair proteins Rad51 and Mre11, has been recently described for the combination VPA-AZD2461, a PARP inhibitor [158].

Low-doses of interferon alpha (IFN-alpha), although ineffective as single agent, profoundly boosted the anti-tumor properties of VPA, reducing tumor cell adhesion, migration, and growth both in vitro and in vivo [159].

Another interesting association between VPA and the cholesterol lowering agent simvastatin was studied in metastatic castration-resistant prostate cancer (mCRPC) cell and animal models. VPA/simvastatin combination sensitized mCRPC cells to docetaxel, a standard of care in mCRCP treatment, and reverted cancer stem cells (CSCs)-driven docetaxel-resistance by modulating the mevalonate-YAP axis [160]. The combination of hydralazine (DNA methylation inhibitor) and HDACi (Panobinostat or VPA) also seems promising [161], while the association of VPA with the multiple receptor tyrosine kinase inhibitor AEE788 did not result in an additive anti-tumor effect and had no advantage over VPA monotreatment in vitro [162].

HDACi are also promising as candidate radiosensitizers for many types of cancers, including PCa. VPA, even at low concentrations, can significantly increase ionizing radiation (IR)-induced apoptosis, very likely by stabilizing a specific acetyl modification (lysine 120) of the p53 tumor suppressor protein and the subsequent modulation of the mitochondrial membrane potential [163]. Similarly, the combined pretreatment with VPA and 1,25-dihydroxyvitamin D3, 1,25(OH)2D3, the active metabolite of vitamin D, a well-known anticancer agent, followed by IR also resulted in an enhanced damaging effect of IR on PCa cells. This will allow for a reduction in IR doses administered to PCa patients, strongly limiting the severity of IR-induced side effects [164].

#### 4.2.5. VPA in PCa Clinical Studies

Only two clinical trials have been conducted using VPA in monotherapy. A Phase II clinical trial tested the safety and efficacy of oral VPA in patients with CRPC [165]. Although PSA levels were inversely correlated with VPA levels, confirming VPA efficacy in a clinical setting, the investigators discouraged the administration of oral VPA since drug bioavailability could not be easily monitored using this formulation, and it was not tolerated well by CRPC patients (neurologic symptoms and fatigue occurred, leading to therapy interruption or dose delays). However, no adverse events (AEs) have been experienced by VPA-treated patients with progressive, non-metastatic PCa, enrolled in another randomized phase II trial, started in 2008 and terminated, on PI decision, in 2018. No results on the efficacy have been published yet [166]. Similarly, no AEs were observed and stable disease (SD) ≥ 6 months was achieved in a phase I study conducted on the combination of VPA and the anti-VEGF monoclonal antibody bevacizumab in patients with advanced cancers, including PCa [167].

### 4.3. Other AEDs in PCa

The AED gabapentin (GBP) is commonly also used as an adjuvant analgesic in the treatment of cancer-associated neuropathic pain [168] and seems potentially useful in the treatment of hot flashes experienced by women in menopause and survivors of BC or PCa receiving estrogen or androgen-deprivation therapies [169], although this role has been recently questioned by the Oncology Nursing Society [170].

Moreover, by binding to the α2δ2 subunit of VGCCs, GBP seems to prevent α2δ2 recycling of the plasma membrane, causing a chronic inhibitory effect on calcium currents [171]. Very likely this is the mechanism that explains the tumor growth inhibition of xenografts deriving from α2δ2-overexpressing LNCaP cells, which results in being more tumorigenic than control LNCaP cells, due to their increased proliferation rate, progression and ability to stimulate angiogenesis through a higher secretion of VEGF [172]. Oddly, Bugan et al. failed to observe any effect on the primary tumor, whereas they report a dose-dependent effect on lung metastasis in a GBP-treated Dunning rat PCa model. In particular, low doses of GBP had no effect on pulmonary metastasis, while intermediate doses reduced small metastasis by over 60%, compared to high doses that increased the metastatic potential by over 100% [173].

The mechanisms involved in PCa metastatic events seem to be related to an increased expression of VGSCs in PCa cells, which are involved in the stimulation of directional motility and metastatic cascade [174]. Indeed, the expression of VGSCs has been associated with the metastatic behavior of PCa, both in vitro and in vivo [175,176,177]. Acting as VGSC blockers, the AED PHT has been shown to reduce the motility index of the highly invasive MAT-LyLu PCa cell line [174]. Moreover, both PHT and CBZ were able to inhibit the secretion of PSA by LNCaP and IL-6 by DU-145 and PC-3 cell lines, as well as to the growth in the matrigel of all three PCa cell lines [178].

In a recent Phase II trial on high-risk, biochemically recurrent, non-metastatic prostate cancer (BRPC), PHT was used only as an adjuvant, together with methoxsalen and sirolimus, to enhance the antineoplastic effects of the amino-acid analogue Racemetyrosine (SM-88). None of the adjuvant treatments had direct antineoplastic effects at the low doses used. No patients developed metastatic disease while on treatment (metastases free survival = 100%). There were no treatment-related AEs, and quality of life (QoL) was unchanged from baseline [179].

### 4.4. AEDs Users and PCa Risk

The inhibitory effect of the AEDs on PSA serum levels in individuals receiving long-term treatment with anticonvulsant drugs, as well as their antiproliferative activity on PCa cells in vitro and in vivo, also accompanied by a significant reduction in mRNA and protein synthesis of PSA, has been related to AEDs potential to reduce PCa risk [180]. Salminen et al. confirmed a decrease in PCa risk among men using AEDs compared to nonuser in a population-based case–control study. A similar PCa risk decrease was observed among users of HDACi AEDs, but no risk difference was found when comparing HDACi AEDs users to users of other AEDs [181]. Nevertheless, very recently, the same authors reported that AEDs use was associated with an increased risk of PCa mortality compared to non-users, likely reflecting the differences between men with epilepsy and those without. Moreover, the use of HDACi AEDs was not significantly associated with decreased PCa mortality compared to the use of other AEDs, although PCa mortality tended to decrease along with the increasing intensity of HDACi AEDs use. Therefore, further studies are needed to elucidate whether this decreasing risk trend depends on the specific AED used [182].

## 5. AEDs in Other Tumor Types

### 5.1. VGICs and HDACs Prognostic and Therapeutic Role in Other Tumors

VGICs and, in particular, VGSCs have been reported to be expressed and promote invasive abilities and progression in cervical [183,184], ovarian [185,186,187], colorectal [30,188,189,190,191,192], gastric [193,194] and non-small cell lung cancer (NSCLC) [195,196], as well as in myeloid leukemia [197] cells.

Similarly, HDACs have also been found aberrantly expressed in ovarian [49,198] and endometrial [199] cancer, NSCLC [200,201], brain tumors [202] and hematologic malignancies [203], including multiple myeloma [204].

Therefore, HDACs and VGICs might be considered potential molecular markers also in all these types of cancer and might be therapeutic targets, explaining the beneficial effects of AEDs.

### 5.2. VPA, Other AEDs and Drug Combinations in Other Tumors

An overview of VPA activity in different tumor types, including ovarian, cervical, gastric and pancreatic cancer, non-small cell lung cancer (NSCLC), hepatocellular carcinoma (HCC) and acute myeloid leukemia (AML), has been recently published by Lipska et al. [205] and Wu et al. [206].

Moreover, Cucchiara et al. proposed a highly detailed and well-documented overview of both preclinical and clinical data related to the anticancer effect of commonly prescribed AEDs (including levetiracetam (LEV), VPA, oxcarbazepine and others) in brain-tumor-related epilepsy (BTRE) [207]. In this context, oxcarbazepine (OXC) and VPA have been previously reported to significantly inhibit proliferation and induce apoptosis in glioblastoma cells. Similarly, the other tested drugs LTG, GBP, PHT or Tiagabine (TGB) promoted growth inhibition, although with less efficiency [208]. A special focus on the inhibitory effects of VPA on glioma, its underlying mechanisms and clinical implications was recently reviewed by Han et al. [209]. LEV, a relatively new AED also used to control seizures during glioblastoma multiforme (GBM) treatment, has been reported to modulate HDAC levels by silencing O6-methylguanine-DNA methyltransferase (MGMT), which, in turn, improves the activity of the alkylating agent temozolomide (TMZ), i.e., the most effective chemotherapy to treat GBM [210]. Interestingly, from a retrospective cohort study, the administration of LEV, if compared to other AEDs, seems to prolong the OS period, but only in the subset of GBM patients with methylated MGMT promoters who are receiving TMZ chemotherapy [211].

Furthermore, emerging evidences indicate that AEDs may increase radiosensitivity, and therefore improve clinical outcomes, in GBM patients [212]. A similar observation was recently described by Lai et al. in murine melanoma B16-F10 cells pre-treated with VPA and then irradiated. The combination treatment significantly inhibited the growth of melanoma cells, increasing DNA DSBs, thus demonstrating that VPA can serve as a radiosensitizer in the treatment of melanoma [213]. VPA has been also shown to exert a dose-dependent growth inhibition and to trigger apoptosis at high doses in G-361 human melanoma cell line [214]. VPA significantly inhibited cellular proliferation and induced apoptosis in PLC/PRF5 human hepatocellular carcinoma (HCC) cell lines [215]. In HeLa cervical cancer cells, VPA inhibited cell growth by increasing ROS levels and inducing GSH depletion [216]. Due to its pro-oxidative potential, VPA also resulted in being cytotoxic to human colorectal adenocarcinoma (HT-29) cells, increasing intracellular ROS and inducing mitochondrial dysfunction and apoptosis-related morphological damage, suggesting a potential use as an adjuvant therapy in colorectal cancer (CRC) [217].

The phospho-VPA (P-V; MDC-1112), a valproic acid derivative, inhibited the growth of human pancreatic cancer (PC) xenografts in mice by 60–97%, and 100% when combined with cimetidine. MDC-1112 inhibited STAT3 signaling, reduced its mitochondrial levels by preventing its translocation from the cytosol and while enhancing the mitochondrial levels of ROS, which triggered apoptosis [218]. With this same underlying mechanism, MDC-1112 was also reported to inhibit the growth of GBM cell lines in a concentration- and time-dependent manner, sparing normal human astrocytes. In vivo, MDC-1112 reduced the growth of subcutaneous GBM xenografts in mice by up to 78.2% vs. controls. Moreover, MDC-1112 extended survival in an intracranial xenograft model [219].

MDC-1112 has been also reported to reduce PC growth by 58% in patient-derived tumor xenografts (PDX), whereas MDC-1112/gemcitabine (GEM) combination reduced tumor growth by 94% [220]. Finally, to improve its efficacy, MDC-1112 was formulated in Poly-(L)-lactic acid-poly(ethylene glycol) (PLLA-PEG) nanoparticles (NPs). PLLA-PEG improved MDC-1112 pharmacokinetics in mice enhancing the blood levels of MDC-1112 [221].

VPA also inhibited the proliferation of SHSY5Y neuroblastoma (NB) cancer cells in a time- and dose-dependent manner by downregulating URG4/URGCP and its transcriptional target CCND1, leading, in turn, to cell cycle arrest [222]. The combination of VPA/IFN-alpha synergistically inhibited NB cell growth in vitro and in vivo [223]. VPA was also able to potentiate staurosporine (STS)-induced apoptosis in NB cells via the downregulation of the expression of Akt and survivin, an anti-apoptotic protein crucial in resistance to STS-mediated cytotoxicity. Interestingly, valpromide (VPM), a VPA analog but devoid of HDAC inhibitory activity, did also potentiate STS-mediated NB cell death, through reduction in survivin and Akt levels, thus suggesting that HDAC inhibition might not be crucial for STS-induced apoptosis, and other mechanisms might be involved [224].

Another interesting combination has been recently described for VPA and Arsenic Trioxide (ATO; As2O3), another anti-cancer agent for various solid tumors and hematological malignancy, which has been reported to efficiently inhibit the growth of lung cancer cells both in vitro and in vivo. Synergistically enhancing ATO anti-cancer effect, the ATO/VPA combination would allow reducing ATO toxicity [225].

The main molecular targets and biological effects of several AEDs in different tumor types are summarized in Table 1.

## 6. Drug Delivery Systems (DDSs) Development to Overcome Toxicity and Low Solubility of AEDs and Their Derivatives

AEDs show acute, although often mild and reversible, dose-related and mainly neurological effects (e.g., sedation, dizziness, blurred vision, diplopia, and tremor), in addition to neurocognitive and psychiatric symptoms (e.g., depression, anxiety, irritability, impaired concentration, mood changes, hyperactivity, and, in rare cases, psychosis). With some exceptions, the newer AEDs seem to be better tolerated than older drugs [226]. Some other effects are idiosyncratic adverse drug reactions (ADRs) caused by the formation of reactive metabolite (RM) after the bioactivation process, which may lead to life-threatening adverse effects or immune-mediated reactions [227]. For these reasons, patients usually discontinue the treatment. A recent review discusses AEDs deriving RMs and the research efforts that have been taken, focusing on various synthetic strategies adopted to minimize AEDs toxicity [227]. For instance, since VPA is notoriously hepatotoxic, N-(2-hydroxyphenyl)-2-propylpentanamide (o-OH-VPA), a VPA aryl derivative, has been designed in silico as a selective inhibitor of HDAC8. o-OH-VPA was much more effective than VPA in reducing cell survival of HeLa cells without exerting toxicity on normal cells [228].

In addition to unwanted toxicity, VPA has limited serum half-life and rapid drug metabolization. To circumvent these problems, the immobilization of VPA in a polysaccharide matrix (cellulose and dextran) was proposed as an effective nanocarrier system, which resulted in being hemocompatible and nontoxic [229]. VPA-loaded NPs, based on cellulose and dextran VPA esters, were further modified with sulfuric acid half ester moieties to improve intracellular drug release. The NPs did not show any toxicity both in vitro and in vivo and were able to induce histone H3 hyperacetylation [230]. A VPA-loaded, green-light-responsive nano-DDS [DAN-bis(HO-Naph-VPA)] was recently developed, showing good cytocompatibility, excellent cellular internalization and effective cancer cell killing ability [231]. Finally, to overcome VPA derivatives’ poor solubility in water, G4 PAMAM, four generations of polyamidoamine dendrimers transporting weakly water-soluble aryl-VPA-derivate compounds, were developed, and their increased efficacy compared the free molecules was confirmed on BC cell lines [80].

## 7. Conclusions

The occurrence of resistance to cancer treatments and the consequent therapeutic inefficacy demands a continuous effort in trying to identify new molecular targets for new potential chemical entities. Although great advances have been made in cancer treatment, current therapies still have several drawbacks, such as limited efficacy, severe side effects and, not least, elevated costs, which are generally due to the high rate of failure of tested molecules in early-stage clinical trials, but also to the expensive developing procedures. Therefore, repurposing already approved drugs to treat off-label diseases, including cancer, might represent an attractive way to lower overall development costs and to shorten development steps.

In this context, several AEDs have been associated with anti-tumor activity in various types of cancers, suggesting that certain actors involved in epileptogenesis may also contribute to tumorigenesis. While the mechanisms through which AEDs perform their anticonvulsant functions are well established, the molecular basis of their anti-cancer effects is mostly ascribed to their ability to inhibit HDACs. However, emerging literature on AEDs is also claiming their role as sodium and calcium currents blockers, VGSCs and VGCCs being aberrantly expressed and often deregulated in various cancers.

Here, we tried to give an accurate overview of the well-established molecular targets and the different mechanism of action exerted by commonly used AEDs on various types of cancer. Very likely, additional and still unveiled targets with potential diagnostic, prognostic and therapeutic roles do exist for this specific class of drugs. This might help explain the plethora of effects observed in the different cancers and listed in this review.

As an example, recent unpublished data from our laboratory clearly suggest the existence of additional molecular targets for LTG, which are currently under investigation.

Finally, although toxicity issues have been raised that would limit AEDs use in monotherapy, combined therapy with other conventional cytostatic drugs would allow the reduction in the doses of both AEDs and chemotherapeutics, thus limiting potential AEs in cancer patients. Alternatively, great attention is being given to the possibility of safely delivering the AED to the tumor site through its loading onto opportunely designed DDSs, especially in the case of water-insoluble molecules.

Therefore, research on widely used and already approved anticonvulsants, especially in a clinical setting, should be strongly encouraged so to ascertain if AEDs could represent a promising and cost-effective therapeutic strategy in cancer management.

## Figures and Tables

**Table 1:** Anticancer activity of several AEDs in different tumor types. Molecular targets, biological effects and main underlying mechanisms are listed.

Tumor Type	AED	Molecular Targets	Study Models	Biological Effects and Main Underlying Mechanisms	References
Breast	VPA and Derivatives	VGSCs HDACi	In vitro and in vivo clinical studies	Inhibit proliferation, cell cycle, survival, migration and hormone receptor expression	[42,76,78,79,81,82,83,86,88,91,92]
	PHT	VGSCs (Na_v_1.5)	In vitro and in vivo	Inhibit cells migration, invasion and metastasis	[14,40,64,94,95]
	LTG	VGSCs VGCCs HDACi	In vitro and in vivo	Anti-proliferative effect and inhibition of breast tumor growth	[14,15,16,17,98,99,100,101,102,103,104]
	CBZ	HDACi CCIDs	In vitro	Anti-metastatic potential by inducing HER2 proteasomal degradation; inhibition of cell proliferation. Inactivation of MLC2, MYPT1 and FAK mobility proteins	[111,112,113]
Prostate	VPA and Derivatives	HDACi C/EBPα/SREBP-1 E-cadherin mTOR	In vitro and in vivo clinical studies	PSA down-regulation; caspase-3 up regulation; cell cycle arrest, apoptosis, autophagy and suppression of tumor angiogenesis. Cell growth Inhibition by reducing lipogenesis. Inhibition of cells migration. Reduction of tumor growth in vivo.	[127,128,131,132,133,134,135,136,137,138,139,140,141,150]
	GBP	Calcium channel α2δ2 subunit	In vitro	Inhibition of tumor cell growth	[171]
	CBZ	VGSCs	In vitro	Reduction of cell motility; inhibition of PSA secretion and of cell growth in matrigel	[173,177]
	PHT	VGSCs	In vitro	Inhibition of PSA secretion and of cell growth in matrigel	[173,177]
Brain	VPA and Derivatives	HDACi URG4/URGCP and CCND1	In vitro and in vivo	Inhibition of proliferation; apoptosis; growth suppression through STAT3 phosphorylation inhibition; cell cycle arrest	[206,207,219,222,223,224]
	OXC	VGSCs	In vitro	Inhibition of proliferation; apoptosis	[207]
	LTG	HDACs PI3k/AKT VGSCs and VGCCs?	In vitro	Inhibition of proliferation; apoptosis	[207,208]
	GBP	VGSCs	In vitro	Inhibition of proliferation; apoptosis	[207,208]
	TGB	GAT-1	In vitro	Inhibition of proliferation; apoptosis	[207,208]
	PHT	VGSCs	In vitro	Inhibition of proliferation; apoptosis	[207,208]
Hepatocellular carcinoma	VPA	HDACi	In vitro	Inhibition of proliferation; apoptosis	[215]
Cervical	VPA	HDACi ROS	In vitro	Inhibition of proliferation via caspase-dependent apoptosis	[216]
Pancreatic	VPA and Derivatives	mitochondrial STAT3	In vitro and in vivo	Inhibition of proliferation; apoptosis	[218,219,221]
Melanoma	VPA	increasing DNA DSBs	In vitro and in vivo	Inhibition of proliferation; apoptosis	[213,214]
